# Specialty grand challenge in neuroinfectious diseases

**DOI:** 10.3389/fneur.2025.1557610

**Published:** 2025-02-14

**Authors:** U. K. Misra

**Affiliations:** T.S. Misra Medical College and Hospital, Apollo Medics Super Speciality Hospital and Vivekanand Polyclinic and Institute of Medical Sciences, Lucknow, India

**Keywords:** infection, fungal meningitis, bacterial meningitis, geriatrics, artificial intelligence, climate change, drug resistance, dengue

## Introduction

The recent surge in infectious disease outbreaks, such as the COVID-19 pandemic, the Zika virus outbreak in South America, Ebola in Africa, Dengue in Asia, West Nile virus in the Middle East and the Americas, and Enterovirus infections in North America, underscores a growing global health challenge. COVID-19 alone resulted in a high mortality rate, with more than 4 million fatalities and morbidity in a third of survivors. Bacterial meningitis remains a major cause of death and disability worldwide. Factors such as climate change, deforestation, and pathogen-related factors are driving these outbreaks (1). Although primarily a respiratory infection, COVID-19 has significant neurological manifestations that include encephalopathy, stroke, neuromuscular disorders, and permanent neurological disability. Central nervous system (CNS) infections are particularly concerning because of their diagnostic complexity and the lack of effective treatments for many pathogens that result in death and disability. CNS infections require multidisciplinary efforts to document, diagnose, and treat them to improve patient outcomes.

## Changing epidemiology of pathogens

### Pathogen X

The term “Pathogen X” refers to a presently unknown infectious agent with pandemic potential that is regarded to emerge through zoonotic spillover, spreading from animal reservoirs to humans (2). While viruses are the most likely neuroinvasive agents for a future pandemic, bacterial and fungal CNS infections also warrant attention. Fungal infections are of particular concern in immunocompromised individuals, while bacterial infections are complicated by antimicrobial resistance.

### Dengue virus (DENV)

Globally, DENV infection is the fastest-spreading vector-borne disease (3). Approximately 1% of patients with DENV infection develop neurological complications, particularly with DENV-2 and DENV-3 serotypes. Neurological manifestations of DENV infection include encephalopathy, encephalitis, immune-mediated syndromes, and myositis, which has been later reported as Dengue-associated transient muscle dysfunction (4). The spectrum of DENV-related neurological manifestations ranges from encephalitis/encephalopathy at one pole to muscle involvement at the other, often with overlapping cases in between (4).

#### Climate change and dengue

Dengue is prevalent in tropical and subtropical regions, with a 30-fold increase in incidence over the past four decades, affecting over 390 million people annually (5). Global warming has expanded the habitat of Aedes mosquitoes, the primary vector of DENV, as higher temperatures, humidity, and precipitation amplify vector populations. The majority of DENV cases (90%) between 1983 and 2001 occurred at temperatures of 27–29.5°C and humidity levels exceeding 75% (6). Dengue outbreaks have been linked to El Niño and La Niña events, which cause fluctuations in temperature and heavy rainfall. Record outbreaks of Dengue were reported in the Americas in 2023, with over three million cases (7), and in Bangladesh, attributed to high temperatures and El Niño (8). Projections estimate that an additional 25 billion people will live in DENV-prone areas by 2080, adding to the four billion already at risk. Rising global temperatures could extend the reach of DENV to new regions, including the USA, Japan, and China, with South and West Africa likely to experience the greatest increase (9). Climate change has also affected Europe and there is a potential for an increase in the occurrence of Zika, Chikungunya and Dengue in the Southwestern region of Europe (10).

### Fungal meningitis

Approximately 300 of the 100,000 known fungi can cause CNS infections. Fungal meningitis is prevalent in immunocompromised patients, and those with nosocomial infections, particularly by Candida, Aspergillus, and Mucor species, are an important concern. Candida is the most common nosocomial fungal infection following neurosurgery.

#### Medical tourism and fungal meningitis

Medical tourism is emerging as a significant risk for fungal meningitis. Historically, patients from nations with poor healthcare systems sought medical treatment in those with advanced healthcare, such as the USA, UK, and Europe. However, this trend has reversed recently, with patients from developed countries seeking treatment in less developed countries because of lower costs, shorter waiting times, and access to specific treatments. In 2017, 1.3 million Americans sought medical care abroad for procedures such as cosmetic surgery, dental treatments, and organ transplants (11). The lack of regulatory oversight in these settings increases the risk of infection. Fungal meningitis has been reported in the United States following contaminated spinal and epidural anesthesia for cosmetic procedures in Mexico (12).

## Drug-resistant meningitis

Acute bacterial meningitis (ABM) is an important cause of mortality and morbidity, with fatality rates of 8%−15% despite treatment and sequelae in 22% of the survivors (13). While Haemophilus influenzae has been controlled by vaccination, *Streptococcus pneumoniae* and *Neisseria meningitidis* remain major pathogens.

Emerging antibiotic resistance threatens progress in the treatment of ABM. Poor CNS penetration of antibiotics necessitates the use of high-dose regimens for the treatment of meningitis. Penicillin-resistant *S. pneumoniae* was first identified in 1967 and caused treatment failures through the 1970s−1990s (14). Third-generation cephalosporins, such as ceftriaxone, have been used as effective alternatives, but resistance to both penicillin and ceftriaxone has been reported, requiring treatment with vancomycin and rifampin (15). Resistance in *N. meningitidis* is less common, but instances of ceftriaxone-resistant strains were also reported in the USA in the 1970s (16). Resistance to penicillin, rifampin, and ciprofloxacin has also been reported (17), posing a challenge in regions like the African meningitis belt, where vaccination efforts have reduced disease incidence but increased the prevalence of non-vaccine serotypes.

## Multidrug-resistant tuberculosis (MDR-TB)

MDR-TB, defined as resistance to isoniazid and rifampicin, is a strong predictor of mortality (18). Resistance arises from primary infection with drug-resistant Mycobacterium tuberculosis or mutations during treatment (19). In Asia, MDR-TB is reported in 5.2% of cases with isoniazid resistance in 9.4% (20). In Europe, MDR-TB occurs in 3.5% of cases, with resistance to at least one drug in 14.1% (21). Mortality rates for MDR-TB range from 16.7% to 100% (22). To address this challenge, the International TBM Consortium is planning a large trial to evaluate improved treatment regimens (23).

Antimicrobial resistance is a natural phenomenon exacerbated by the widespread use of antibiotics. In the United States, 30% of outpatient antibiotic prescriptions were deemed inappropriate in 2010–11 (24). Global antibiotic consumption increased by 65% between 2000 and 2015, driven primarily by low- and middle-income countries (LMICs). Urbanization and increased transportation in LMICs are projected to increase antimicrobial resistance by more than 50% in some regions. Poor surveillance and infrastructure exacerbate community-level transmission of resistant organisms through wastewater and food processing systems (25).

Antimicrobial use in agriculture also contributes significantly to resistance. In 2017, 73% of antibiotics were used in animals (26). Industrial farming often employs antimicrobials to promote growth and longevity in livestock (27). Drug-resistant organisms can persist in soil and manure, contaminating water sources used for drinking and sanitation (28). Contaminated food further facilitates the spread of resistance through direct consumption or during processing.

## Geriatric infections

The human population is aging much faster than in the past. The number of people aged 60 years and older is projected to increase by 60% in developed countries and 160% in less developed countries over the next 30 years (29). By 2050, one-third of the world's population will be over 60 years old, with 80% residing in LMICs. Infectious diseases remain one of the five leading causes of death and one of the 10 leading reasons for hospitalization among individuals aged 65 years or older (30).

The aging population brings specific challenges regarding infection, leading to significant morbidity and mortality. Associated comorbidities such as diabetes, hypertension, stroke, cancer, malnutrition, alcoholism, immobility, institutionalization, and age-related immune decline, along with economic factors, further increase the risk of severe infections. Aging often modifies the clinical presentation of infections, causing delays in seeking medical care and hospitalization. The majority of infections occur in resource-poor and tropical countries, underscoring the need for cost-effective medical solutions to ensure that advancements reach those who need them most. There is a need for modifications in medical education and resources to deal with aging populations and infections.

## Cost-effective medicine

Economic incentives are a key driver of medical research and development, making modern medicine expensive and often inaccessible in resource-poor settings. Providing cost-effective solutions is essential to ensure affordable treatment without compromising outcomes. The high costs of intensive care unit (ICU) management can be mitigated by rationalizing admission policies, avoiding unnecessary investigations, selecting appropriate antibiotics, and reducing errors through better training and education.

The resource crunch in LMICs triggers innovation and sometimes desperate measures, such as the use of prolonged AMBU ventilation for 18 days for respiratory paralysis in Guillain-Barré Syndrome (31), the use of single breath count as a surrogate marker for arterial blood gas changes and as a guide for intubation in Guillain-Barré Syndrome (32), and employing a syndromic approach to Acute Encephalitis Syndrome (AES).

Acute encephalitis syndrome is a critical public health problem in which patients present with acute onset of fever with alteration in consciousness ranging from stupor to coma, with or without new-onset convulsions, excluding simple febrile seizures. A syndromic approach to AES categorizes patients into those with primary CNS involvement (e.g., Japanese encephalitis, herpes simplex encephalitis, West Nile virus encephalitis) and those with systemic features (e.g., rash, myalgia, thrombocytopenia, hypotension, and hepatic or renal dysfunction). Systemic AES may be caused by cerebral malaria, scrub typhus, dengue, chikungunya, or leptospirosis. Neurological AES may benefit from cranial CT/MRI, which reveals characteristic features in diseases like Japanese encephalitis (JE) and herpes simplex encephalitis.

MRI is valuable in patients with neurological AES. If frontotemporal involvement is detected, it suggests herpes simplex encephalitis and acyclovir should be administered, whereas thalamic, basal ganglia or brainstem involvement is suggestive of JE or arboviral encephalitis, and acyclovir may be withheld. For systemic AES, treatable causes should be prioritized. This strategy has helped reduce the cost of AES diagnosis and treatment in Northern India (33). Protocols tailored to specific regions and etiologies may significantly improve outcomes at a lower cost. Research on major health issues in LMICs such as on cost-effective medicine through research funding, grants, publication promotion, and budget allocation can go a long way in improving the medical care in the areas where it is most needed.

## Diagnostics

Advancements in neurodiagnostics have revolutionized the field of neuro-infection, but many infections remain undiagnosed (34, 35). While polymerase chain reaction (PCR) and multiplex PCR are accessible in resource-rich settings, these are often unavailable in low-resource settings. Hypothesis-free diagnostics using sequencing techniques have emerged for the diagnosis of CNS infections, providing unbiased results by analyzing all non-human DNA in a sample. This method has identified pathogens that were previously diagnosable only via biopsy or autopsy. Real-time quaking-induced conversion (RT-QIC) has improved cerebrospinal fluid testing for prion diseases (36).

MRI has significantly improved the diagnosis of CNS infections and will continue to do so with wider availability and technical advancements. Infections cause inflammation, which increases tissue density, while cell death reduces it on MRI. Machine learning (ML) and artificial intelligence (AI) protocols are now being used to detect infections through medical imaging of the lungs (37). While these methods primarily identify secondary effects rather than pathogens, bacteria-specific PET tracers are being evaluated, although these remain experimental (38–40).

## Artificial intelligence (AI)

Artificial intelligence is advancing rapidly, contributing to anti-infective drug discovery, understanding of infection biology, and the development of new diagnostics. Generalisable AI models require large and representative datasets, including data from LMICs and remote areas. Programmable datasets, such as nucleic acid and amino acid sequences, are being integrated into ML models to predict drug efficacy, host-pathogen interactions, and host responses. These advancements are expected to aid in the design of next-generation drugs, vaccines, and diagnostics for infectious diseases (41).

The AI model for diagnostic prediction may have inaccuracies. A recent study evaluating the diagnosis and outcome of COVID-19 patients based on chest radiographs and CT scans revealed poor predictive ability of the model because of methodological flaws and /or bias (42). Obtaining high-quality data relevant to new or emerging pathogens or strains, especially in LMICs may be difficult because of a lack of infrastructure and public health resources. ML models based on limited data may result in bias and misdiagnosis, limiting their application in clinical settings.

The application of AI in real-world settings, however, poses many challenges. Ethical and practical issues must be addressed before AI can be widely applied to infectious diseases. Clinical studies, regulatory frameworks, and reporting standards are needed. While AI is unlikely to replace clinicians or academics in the near future, over-reliance on AI could lead to the deskilling of physicians. AI may struggle to adapt to novel situations or individual patients as effectively as human clinicians, particularly non-specialists or general practitioners.

## Conclusions and way forward

The COVID-19 pandemic demonstrated that every country is vulnerable to public health emergencies and highlighted the need to develop and implement a coherent and context-specific strategy to deal with future emergencies. A primary healthcare approach that provides universal and equitable promotive, preventive, and curative services through whole-of-government and whole-of-society approaches, is essential.

Reducing infections globally requires several short-term and long-term measures. Short-term measures include rational use of antibiotics, cost-effective medicine, vaccination, hygiene, surveillance, monitoring, quarantine, and isolation. Long-term strategies include education, sanitation, prevention of environmental damage, improvement of health policy frameworks, socioeconomic development, and meaningful research.
